# Tuberculosis in the Caribbean: using spacer oligonucleotide typing to understand strain origin and transmission.

**DOI:** 10.3201/eid0503.990311

**Published:** 1999

**Authors:** C Sola, A Devallois, L Horgen, J Maïsetti, I Filliol, E Legrand, N Rastogi

**Affiliations:** Institut Pasteur de Guadeloupe, Pointe à Pitre.

## Abstract

We used direct repeat (DR)-based spacer oligonucleotide typing (spoligotyping) (in association with double-repetitive element polymerase chain reaction, IS6110-restriction fragment length polymorphism [RFLP], and sometimes DR-RFLP and polymorphic GC-rich sequence-RFLP) to detect epidemiologic links and transmission patterns of Mycobacterium tuberculosis on Martinique, Guadeloupe, and French Guiana. In more than a third of the 218 strains we typed from this region, clusters and isolates shared genetic identity, which suggests epidemiologic links. However, because of limited epidemiologic information, only 14.2% of the strains could be directly linked. When spoligotyping patterns shared by two or more isolates were pooled with 392 spoligotypes from other parts of the world, new matches were detected, which suggests imported transmission. Persisting foci of endemic disease and increased active transmission due to high population flux and HIV-coinfection may be linked to the recent reemergence of tuberculosis in the Caribbean. We also found that several distinct families of spoligotypes are overrepresented in this region.


### Tuberculosis in the Caribbean: Using Spacer Oligonucleotide Typing to Understand Strain Origin and Transmission

**Christophe Sola, Anne Devallois, Lionel Horgen, Jérôme Maïsetti, Ingrid Filliol, Eric Legrand, and Nalin Rastogi**
Institut Pasteur de Guadeloupe, Pointe à Pitre, Guadeloupe

We used direct repeat (DR)-based spacer oligonucleotide typing (spoligotyping) (in association with double-repetitive element–polymerase chain reaction, IS*6110*-restriction fragment length polymorphism [RFLP], and sometimes DR-RFLP and polymorphic GC-rich sequence-RFLP) to detect epidemiologic links and transmission patterns of *Mycobacterium tuberculosis* on Martinique, Guadeloupe, and French Guiana. In more than a third of the 218 strains we typed from this region, clusters and isolates shared genetic identity, which suggests epidemiologic links. However, because of limited epidemiologic information, only 14.2% of the strains could be directly linked. When spoligotyping patterns shared by two or more isolates were pooled with 392 spoligotypes from other parts of the world, new matches were detected, which suggests imported transmission. Persisting foci of endemic disease and increased active transmission due to high population flux and HIV-coinfection may be linked to the recent reemergence of tuberculosis in the Caribbean. We also found that several distinct families of spoligotypes are overrepresented in this region.

Sequencing of the *Mycobacterium tuberculosis* H37Rv genome (1) has facilitated the study of the biodiversity of *M. tuberculosis* around the world (2). We investigated *M. tuberculosis* epidemiology and biodiversity in the Caribbean region, where sequencing data are not available. Caribbean islands possess independent and shared characteristics that justify investigation of both the molecular epidemiology (3) and the evolutionary history of *M. tuberculosis* (4). We used spacer oligonucleotide typing (spoligotyping), double-repetitiveelement (DRE)– polymerase chain reaction (PCR) and IS*6110*-restriction fragment length polymorphism (RFLP) (5-7) to understand the molecular epidemiology and reconstruct the phylogeny of *M. tuberculosis*. The first step was to demonstrate that the chosen molecular methods differentiated isolates with respect to their molecular clonality. The second step was to demonstrate that under proper epidemiologic circumstances, clonality implies active transmission. To eliminate sample bias, we used all data for Guadeloupe during a 3-year period (1994-96) and for Martinique and French Guiana during a 2-year period (1995-96).

*M. tuberculosis* may have developed through the domestication of cattle, and if so, *M. bovis* was its most probable precursor (8). On the basis of restricted allelic diversity, the speciation of *M. tuberculosis* is estimated to have occurred 15,000 to 20,000 years ago (4). It is thought that the spread of tuberculosis (TB) began in Europe around the middle ages and reached the new world in the 16th century, Africa in the 19th century, and only recently, remote regions such as Papua-New Guinea (mid-20th century) and the Amazon (last quarter of the 20th century) (8). The study of the genetic biodiversity of *M. tuberculosis* might help reconstruct this evolutionary scenario. Previous work from our laboratory showed that a significant proportion of patients had conserved (or ancient) strains of

Address for correspondence: Nalin Rastogi, Unité de la Tuberculose & des Mycobactéries, Institut Pasteur, Morne Jolivière, B.P. 484, F-97165 Pointe à Pitre-Cedex, Guadeloupe; fax: 590-893-880; e-mail: rastogi@ipagua.gp.

tubercle bacilli (6), and analysis based on multiple genetic markers showed genetic relatedness between some clusters of bacilli within Guadeloupe (9).

### Demographics and Molecular Typing Methods

Guadeloupe, Martinique, and French Guiana have major demographic differences (Table 1). Guadeloupe and Martinique are densely populated islands in the center of Lesser Antilles; they have similar population sizes (417,000 and 388,000) and areas (1,705 $km^2$ and 1,100 $km^2$) and homogeneous populations (93% French nationals). French Guiana is sparsely populated (150,000), its inhabitants scattered throughout a large territory (91,000 $km^2$). Guadeloupe and Martinique are characterized by an insular population, whereas French Guiana, which borders Surinam and Brazil, is populated by persons of diverse geographic and ethnic origin (many immigrants from South and Central America). The distribution of TB cases among these three French overseas territories reflects their individual demographics. In Guadeloupe and Martinique, 27% and 25% of all TB cases, respectively, were imported, while in French Guiana, 68% of all cases were imported (10). The basic epidemiologic data (incidence, age, sex, nationality, HIV coinfection) for all TB cases reported to health authorities describe a 3-year period (1994-96) (Table 2).

Of 136 TB cases reported in Guadeloupe, 107 had smear- or culture-positive isolates, with culture available in 100 cases; we typed 95 (95%) of 100 isolates. Of 52 cases reported in Martinique, 41 had smear- or culture-positive isolates, and a culture was available in 34 cases. We typed 31 (91%) isolates. Of 124 cases reported in French Guiana, 96 had culture- or smear-positive isolates, with a culture available in 76 cases; we typed 73 (96%) of 76 isolates, along with three that grew in 1995 but came from pathologic specimens received in 1994.

The isolates were first studied by spoligotyping (11), which is based on polymorphism of the DR locus of *M. tuberculosis* (12); spoligotyping results were analyzed by using the Recognizer and Taxotron software (Institut Pasteur, Paris). The 1-Jaccard Index was calculated, and strains were compared by the unweighted pair-group method using arithmetic averages (UPGMA) (13) or by the neighbor-joining method (14). Secondary typing was performed by DRE-PCR (15) or IS*6110*-RFLP (Table 3) (5). To determine clonality between isolates, we used the following algorithm: strains were considered clonal only when a combination

Table 2. Epidemiologic and clinical data for 410 tuberculosis (TB) cases reported to the health authorities from 1994 to 1996 in the French West Indies

| | Guadeloupe | Martinique | French Guiana | Total |
|---|---|---|---|---|
| TB cases | 136 | 89 | 185 | 410 |
| Pleuropulmonary cases | 115 | 71 | 156 | 342 |
| Total TB incidence[a] | 11 | 8.2 | 40.6 | 14.20 |
| Sex ratio | 1.7 | 1.9 | 1.5 | 1.65 |
| PDR to INH (%) | 4.4 | 2.2 | 3.5 | 3.4 |
| PDR to RIF (%) | 1.4 | - | - | - |
| Foreign-born TB patients (%) | 24.5 | 20.3 | 68 | 43.2 |
| TB-HIV coinfection (%)[b] | 28 | 19 | 25 | 24.6 |

[a]New cases/100,000 inhabitants, 1994 to 1996.
[b]Total number of known HIV-positive cases among TB patients was 101. However, foreign-born patients represented 50% of all the TB-HIV coinfected patients in Martinique, 60% in Guadeloupe, and 80% in French Guiana. The % shown is a minimal estimation as HIV serology results were available only for 49% of patients in Martinique, 75% in Guadeloupe, and 80% in French Guiana.
PDR, primary drug resistance; INH, isoniazid; RIF, rifampin.

Table 1. Demographic characteristics of Guadeloupe, Martinique, and French Guiana

| Data | Guadeloupe | Martinique | French Guiana | Total |
|---|---|---|---|---|
| Surface area ($km^2$) | 1,705 | 1,100 | 91,000 | 93,805 |
| Population (1990) | 387,034 | 359,579 | 114,808 | 861,421 |
| Population (1996) | 422,290 | 388,340 | 151,780 | 962,410 |
| Density (inhabitants/$km^2$) | 248 | 353 | 2 | 10 |
| Birth rate/1,000 inhabitants | 16.8 | 14.4 | 29.2 | 17.8 |
| AIDS[a] | 700 | 383 | 561 | 1,644 |
| HIV-positive (%)[b] | 1.4 | 0.8 | 1.8 | 1.3 |

[a]Cumulated cases on June 30, 1996.
[b]On the basis of 7,087 serologic tests performed in departmental clinics in 1994.

Table 3. Molecular fingerprinting and epidemiologic information from spoligotyping-defined clusters shown in Figure 7.[a]

| Spoligo-type no. meth. 1 | No. of strains harboring this type | No. of strains typed by IS*6110* RFLP meth.2 | DR RFLP meth.3 | DRE PCR meth.4 | Summary of results obtained by molecular typing methods[b] | Clinical and epidemiologic data, origin, observations |
|---|---|---|---|---|---|---|
| 1 | 2 | 2 | 2 | 2 | Different by meth. 2, Beijing IS*6110* patterns | Found in Surinam and Guadeloupe |
| 2 | 9 | 9 | 2 | 8 | No subcluster by PGRS-RFLP: all strains identical by meth. 2 and 4 IS-type J and by PGRS-RFLP | 2 patients from hospital A and 2 from B + 2 patients with same surnames |
| 3 | 3 | 3 | 1 | 3 | 2/3 strains identical by meth. 2, 3, and 4, IS-type P | Found in Surinam and Guadeloupe, 2 patients from hospital A |
| 5 | 2 | 1 | 1 | 2 | 2/2 strains identical by meth. 2 and 4, IS type not yet defined | Found in Martinique and Guadeloupe, no evident epidemiologic link |
| 12 | 2 | 2 | 2 | 1 | One spacer difference with type 14, identical by meth. 2 and 3, IS-type A | Very common pattern, represent both active transmission and reactivation |
| 13 | 2 | 2 | 2 | ND | One spacer difference with type 14, identical by meth. 2 and 3, IS-type A | Suspicion of cross-contamination (sampling in the same hospital in 3 days) |
| 14 | 15 | 15 | 13 | 8 | Identical by meth. 2 and 3, identical by method 4 (one band), IS-type A | Very common pattern, represent both active transmission and reactivation |
| 15 | 2 | 2 | 2 | 2 | 2/2 strains identical by meth. 2, 3, and 4, IS-type C | Imported cluster (Surinam or Dominican Republic) |
| 17 | 6 | 6 | 1 | 5 | Subclustered by PGRS-RFLP: 17B, 2 strains identical by meth. 2, 3 and 4, IS-type N | 17B found in 2 Guadeloupean patients hospitalized in same hospital B |
| 29 | 5 | 5 | 4 | 5 | 5/5 strains identical by meth. 2, 3, and 4, IS-type B | 3 of 5 Guadeloupean patients hospitalized in same hospital B |
| 30 | 2 | 2 | 2 | 2 | 2/2 strains identical by meth. 2, 3, and 4 | 2 patients from the same part of Guadeloupe |
| 31 | 2 | 2 | 1 | 1 | 2/2 strains different by meth. 2 | Found in French Guiana and in Guadeloupe, no epidemiologic link |
| 33 | 2 | ND | ND | 2 | 2/2 strains different by meth. 4 | Found in French Guiana, no epidemiologic link |
| 34 | 2 | 2 | 1 | 2 | 2/2 strains identical by meth. 4, method 2: inconclusive, under investigation | Patients from French Guiana, suspected to be epidemiologically linked |
| 42 | 3 | 3 | 1 | 3 | 3/3 strains different by meth. 2 and 4 | Found in Guadeloupe (one patient) and French Guiana (2 patients), no link |
| 44 | 2 | 2 | ND | 2 | 2/2 strains identical by meth. 2 and 4 | 2 patients from St. Maarten (couple) |
| 45 | 6 | 1 | ND | 4 | 4/6 strains identical by meth. 4. (2: pending) | 5 patients from Martinique, one from Guadeloupe, under investigation |
| 46 | 3 | 1 | ND | 3 | 2/3 strains identical by meth. 4 | Found in a Martinique and a Guadeloupe patient, under investigation |
| 50[c] | 29 | 16 | 8 | 20 | Subclustered by meth. 2, 4 and PGRS-RFLP, IS-type E (3 pat.) and F (2 pat) | Imported clusters (Haïti), other links under investigation |
| 51 | 4 | 3 | 2 | 3 | 2/4 strains identical by meth. 2 and 4 (2: pending) | Imported cluster (Haïti) for 2 patients, other links under investigation |
| 53[c] | 29 | 10 | 8 | 20 | Subclustered by meth. 2, 4 and PGRS, IS-type K (2 pat.) and T (3 pat.) | Patients from cluster T come from the same ward of hospital A, 1996 |
| 61 | 2 | 2 | 1 | 1 | 2/2 strains identical by PGRS-RFLP, IS results: under investigation | Found in French Guiana in Guadeloupe, under investigation |
| 63 | 2 | 2 | 1 | 2 | 2/2 strains identical by meth. 2 and 4, IS-type R | 2 patients from hospital A in Guadeloupe, under investigation |
| 64 | 1 | 1 | ND | 1 | *** | 2 isolates from one single patient |
| 65 | 2 | ND | ND | ND | (Results pending) | Found in French Guiana, under investigation |
| 66 | 2 | 2 | ND | 2 | 2/2 strains identical by meth. 2 and 4 | Found in French Guiana, same surname |
| 67 | 2 | 1 | ND | 2 | 2/2 strains identical by meth. 2 and PGRS-RFLP | Found in French Guiana, under investigation |
| 68 | 2 | 1 | ND | ND | (Results pending) | Found in one Martinique patient and in Barbados, under investigation |

[a]Of 218 isolates typed, 145 isolates were grouped in 27 distinct spoligo-defined clusters, which were further analyzed by one or more typing methods—IS*6110*-RFLP (meth. 2), DR-RFLP (meth. 3), and DRE-PCR (meth.4), and sometimes PGRS-RFLP when DRE-PCR or IS*6110*-RFLP results were inconclusive or unavailable.

[b]Isolates with matching spoligotypes and matching IS*6110* patterns (meth. 1 and 2) or with matching spoligotypes and matching DRE-PCR patterns (meth. 1 and 4) were considered to make up a cluster of epidemiologically associated strains.

[c] Noninformative spoligotype patterns that lack any discriminating power.

*** Two clinical isolates from a single patient.

RFLP, restriction fragment length polymorphism; DR, direct repeat; DRE, double-repetitive element; PCR, polymerase chain reaction; PGRS, polymorphic GC-rich probe; ND, not done.

of spoligotyping plus IS*6110*-RFLP or DRE-PCR indicated they were identical. Data from DR-RFLP (12) and *Sma*I-RFLP that used a polymorphic GC-rich probe (PGRS) (16) and available epidemiologic information are also included in Table 3.

### TB Epidemiology

Epidemiologic data for the three territories have been described in detail (Table 2) (10). The mean incidence of cases per 100,000 inhabitants for the period studied was 11 in Guadeloupe, 8.2 in Martinique, and 40.6 in French Guiana. The sex ratio varied from 1.5 in French Guiana to 1.9 in Martinique. In each territory, the highest proportion of TB cases was in persons more than 65 years old; most were reactivated infections. The highest number of cases was in adults 25 to 44 years old; most were new cases due to active disease transmission and the high rate of TB-HIV coinfections. Pleuropulmonary disease was most common (80% to 85% of cases), and drug resistance was rare; primary resistance to isoniazid was 2.2% to 4.4%; single drug resistance to rifampin was not observed in Martinique and French Guiana and was limited to 1.4% of cases in Guadeloupe. Four cases of multidrug-resistant TB (MDR-TB, defined as resistance to at least isoniazid and rifampin) were diagnosed; these cases of secondary drug-resistance were caused by noncompliance to standard antituberculosis chemotherapy. In addition, the rate of TB-HIV coinfection in these three territories was high (19% of total TB cases in Martinique, 25% in French Guiana, and 28% in Guadeloupe). Coinfected patients were most likely men 25 to 44 years of age and of foreign origin (1 of 2 TB-HIV coinfected patients was a foreign national in Martinique, compared with 6 of 10 in Guadeloupe and 8 of 10 in French Guiana). These figures agree with the epidemiologic data concerning the distribution of patients on the basis of their nationality (Figure 1).

In the last 20 years, TB incidence has decreased considerably in these territories. For example, the incidence of new cases in Guadeloupe, 36 per 100,000 inhabitants in 1975, declined to 10 per 100,000 inhabitants in the late 1980s. If the same trend continued, TB would vanish by 2000; however, this decline has slowed and incidence has remained at 10 to 12 per 100,000 since 1989. This reversal of decline is linked to poor economic conditions, increase in unemployment, increase in drug and alcohol abuse, and persistent immigration from countries with a high incidence of TB and ongoing HIV epidemics.

### The Spoligotype Database

To define predominant genotypes and trace the origin of strains and their potential movement, we compared spoligotypes of Caribbean isolates with those of other geographic regions. We built a database of 610 spoligotypes (218 from our own investigation and 392 from other countries) with Excel software. The database contains 167 patterns from Goyal et al. (17); 118 from Kamerbeek et al. (11); 106 from

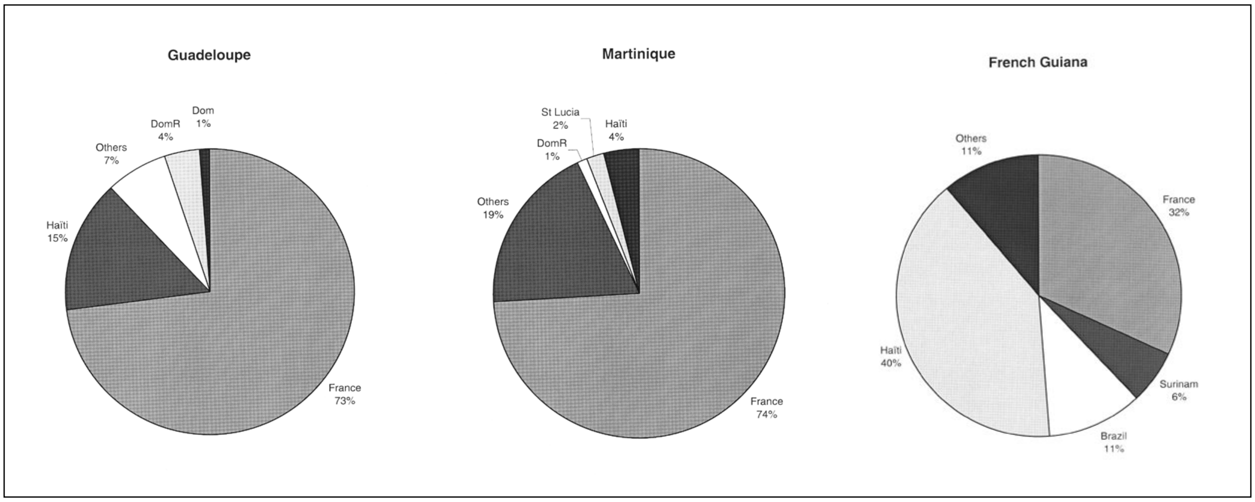

Figure 1. Distribution of tuberculosis cases reported to health authorities in Guadeloupe, Martinique, and French Guiana, 1994-96, by nationality. DomR = Dominican Republic; Dom = Commonwealth of Dominica.

Goguet et al. (18); and a single pattern, named the Manila family, from Douglas et al. (19). In this database, 69 spoligotypes shared by more than two patients in any region of the world were numbered types 1 to 69 (Figure 2): types 1-54 denote 395 spoligotypes that were initially ordered from empty to full squares read from left to right, and types 55 to 69 represent 15 new shared types in chronologic order and correspond to 215 spoligotypes investigated later. The nomenclature is temporary until international guidelines are established. Fourteen shared types might be specific for the Caribbean and bordering Central American regions (patterns 5, 12, 13, 14, 15, 17, 29, 30, 54, 63, 64, 66, 67, 68 [bolded in Figure 2]); 23 shared types were found both in the Caribbean region and in other parts of the world (in regular font in Figure 2). The remaining 32 patterns were not present in the Caribbean (highlighted by an asterisk in Figure 2).

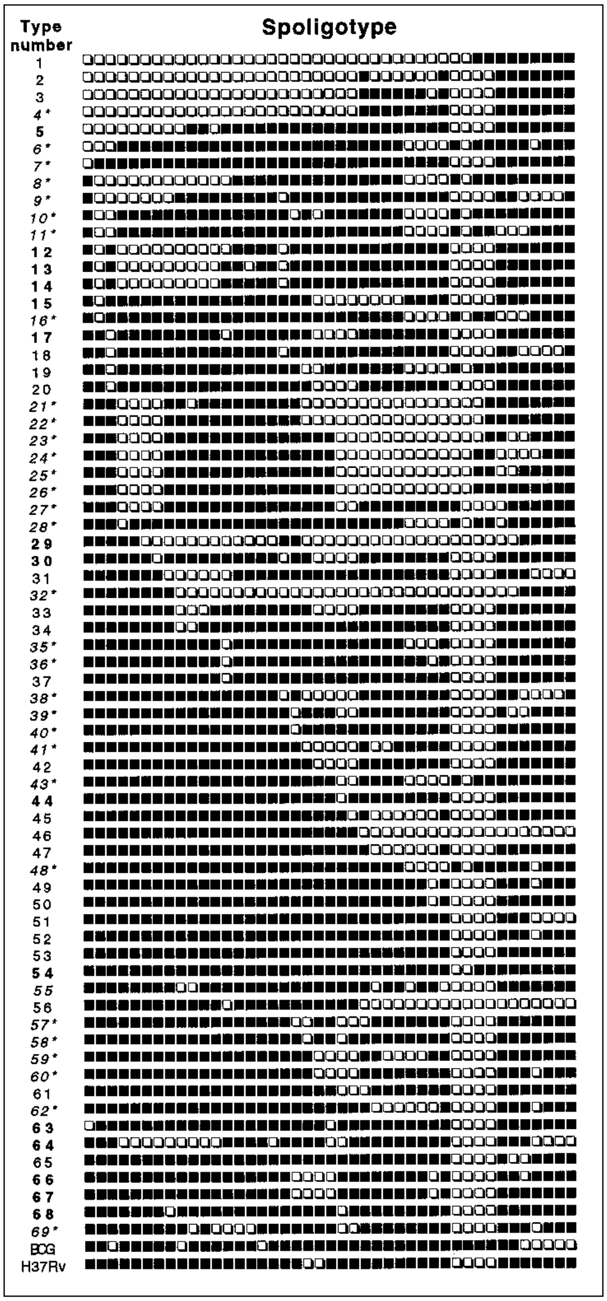

Figure 2. Nomenclature of the spoligotype database (from 1 to 69) based on published spoligotypes (n = 393) and on spoligotypes generated during this investigation (n = 218). The corresponding hybridization patterns for oligonucleotides 1 to 43 (black square, hybridizing; empty square, nonhybridizing) are shown. Type 1 is unique for the Beijing type pattern, whereas type 69 is unique for the Manila type pattern. Bold characters illustrate patterns that have so far been noticed only in Caribbean and neighboring Central American isolates (specific types). Unbolded characters illustrate patterns common to those reported elsewhere (ubiquitous types), whereas italicized characters with an asterisk illustrates patterns not yet found in the Caribbean. BCG and H37Rv spoligotypes are shown as controls.

### Population Structure of *M. tuberculosis* in Guadeloupe

Of 95 isolates from Guadeloupe (Figure 3), 34 were a unique spoligotype, and 61 shared 13 patterns (six patterns were shared by only two isolates, and seven patterns were shared by the remaining 49 [3 to 13 isolates per pattern]). Patterns 2 (4 isolates), 14 (13 isolates), 29 (5 isolates), 50 (10 isolates), and 53 (12 isolates) were the major shared patterns from Guadeloupe and made up 72% of clustered isolates. The interpretation of the population structure from Guadeloupe shows the presence of important nodes on the dendrogram. As illustrated in Figure 3 (from bottom to top), three distinct patterns show strain 95076 (designated type 1 in our database and identical to the Beijing type commonly found in Asia [20]), the recently reported pattern 3 (11), and pattern 2 (18), respectively. Above the patterns 1-3 (pattern 1 is not shown) lies the previously undescribed pattern 29, which might be specific to our region. Two very different groups can be seen on the next node (the "lower" and "upper" groups). The "lower" group, which comprises 20 isolates and two shared patterns (17 and 30), shows a stepwise polymorphism. This group contains

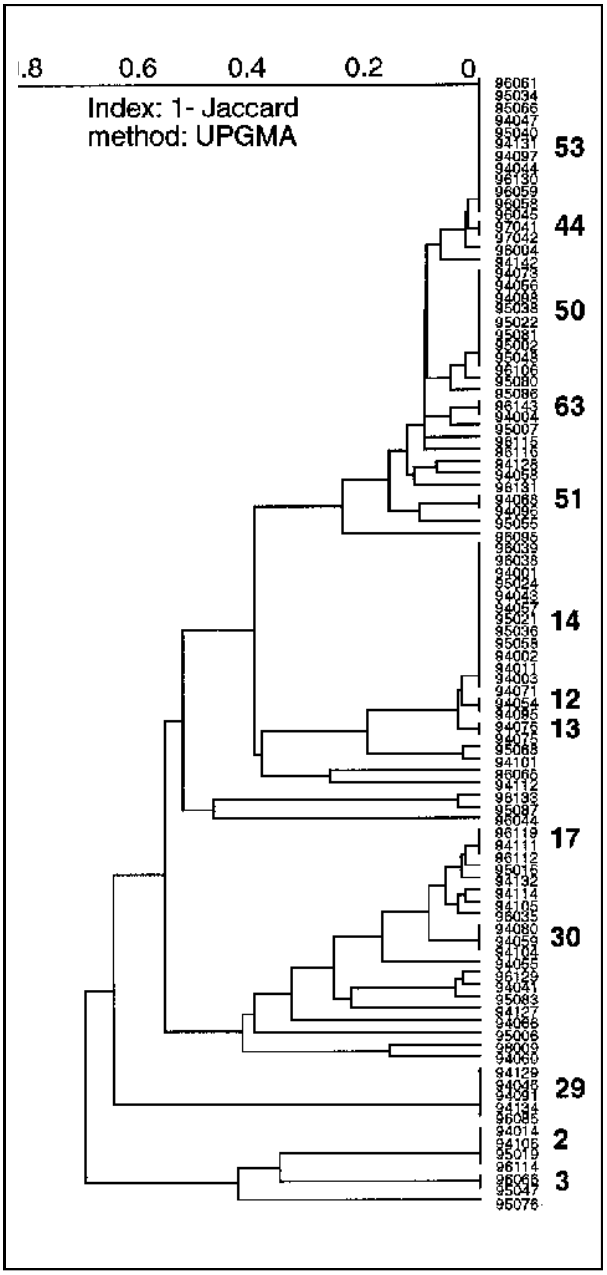

Figure 3. A dendrogram represents spoligotyping results of 95 *Mycobacterium tuberculosis* isolates from Guadeloupe (shared patterns are shown by bold characters). From top to bottom; type 53, ubiquitous; type 44, described by Goguet et al. (two cases) (18); type 54, which does not appear, is found only in isolate 94142, a pattern also found in French Guiana; type 37, which also does not appear, described by Kamerbeek et al. (three cases) (11); type 50, ubiquitous; type 63, specific; type 61, which does not appear is found only in isolate 96131, a pattern also found in Barbados and French Guiana; type 51, described by Goguet et al. (one case) (18); isolate 96095, a *M. bovis* BCG clinical isolate; types 12 to 14, specific types; type 5, which does not appear, is found only in isolate 95068, a pattern also found in Martinique; type 31, which does not appear, is found only in isolate 94112, a pattern also found in French Guiana and already reported by Goguet et al. (two cases) (18); type 46, which does not appear, is found only in isolate 95087, a pattern also found in Martinique and was already reported (18); type 17, specific; type 30, specific; types 45 and 15, which do not appear, are found only for isolates 96129 and 94127, respectively, and share patterns with isolates in Martinique and Surinam; type 29, specific; type 2, previously reported by Goguet et al. (one case) (18); type 3, also found in Surinam, has been described by Kamerbeek et al. (one case) (11); type 1, which does not appear, is found only in 95076, a pattern also found in Surinam, and described (20) as the Beijing type.

many ubiquitous patterns (reported from at least three geographic regions) shared by a variety of isolates from around the world and concerns isolates 95016, 94105, and 94041 of shared types 20, 42, and 47, respectively. The "upper" group, which comprises the remaining 63 isolates and eight shared spoligotypes (types 12, 13, 14, 51, 63, 50, 44, 53), can be further divided into two subgroups: "upper ubiquitous," which lies above *M. bovis* BCG strain 96095 and the "upper specific," which lies below the BCG strain (Figure 3). The "upper specific" subgroup is homogeneous (shared types 12, 13, 14, and strain 95068 of shared type 5) and probably has been present in Guadeloupe for a long time (except in a single isolate with pattern 5 from the neighboring island of Martinique). The "upper ubiquitous" subgroup seems considerably closer to *M. bovis* BCG than do other isolates of *M. tuberculosis* from Guadeloupe.

### Population Structure of *M. tuberculosis* in French Guiana

The first population structure of *M. tuberculosis* isolates from French Guiana is shown in Figure 4. Of 76 isolates, 30 had a unique spoligotype. The remaining 46 shared a total of 11 patterns: eight patterns (51, 33, 17, 42, 67, 66, 65, 34) were shared by only two isolates and three major patterns (pattern 2, 4 isolates; pattern 53, 9 isolates; pattern 50, 17 isolates) were shared by the remaining 30 isolates. The three major patterns represented 65% of clustered isolates in French Guiana. Most clusters were common to those found in Guadeloupe, as well as other regions of the world (types 2, 17, 33, 34, 42, 50, 51, 53, 65), except two patterns that have been so far only reported from French Guiana (types 66, 67). No isolate was of the Beijing type, which is unexpected, considering the number of persons of Chinese origin in French Guiana. A high degree of heterogeneity was observed in French Guiana, which is not surprising given the large surface area, high number of immigrants and persons of various ethnic origins, and high TB incidence rate.

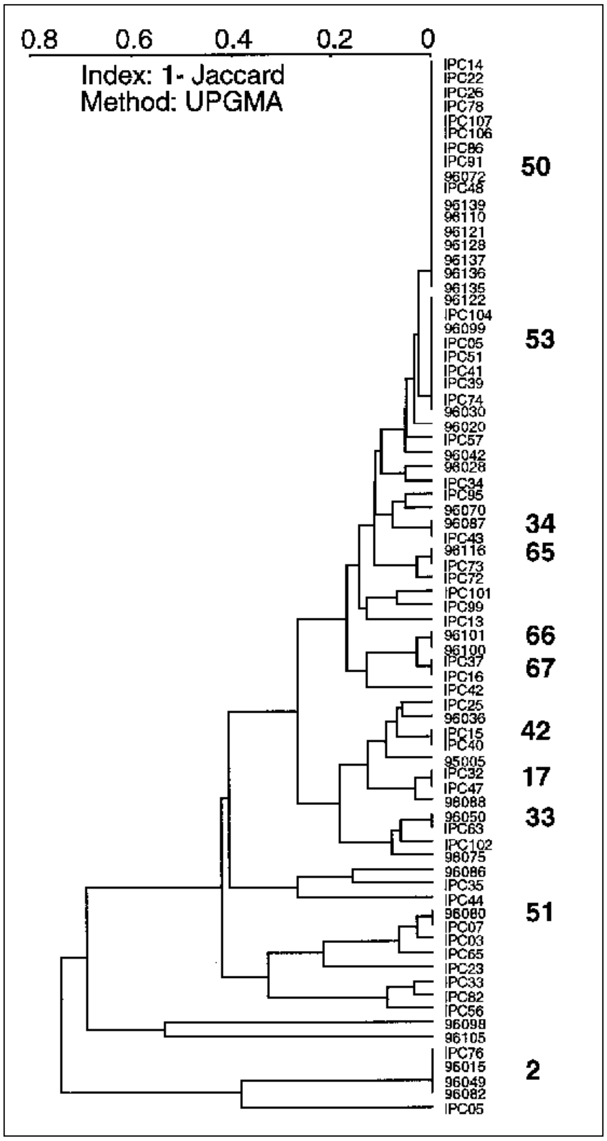

Figure 4. A dendrogram illustrating spoligotyping results of 76 *Mycobacterium tuberculosis* isolates from French Guiana (shared patterns are shown in bold). Top to bottom; patterns 50 and 53, ubiquitous; types 54 and 36, which do not appear, are found only for isolates IPC99 and IPC57, respectively; type 34, a ubiquitous type that was limited to French Guiana in this study; types 66 and 67, specific; type 42, ubiquitous; type 17, specific; type 33, ubiquitous; type 51, ubiquitous; type 31, which does not appear, is found only for isolate IPC23 and is shared with 94112 in Guadeloupe; type 2, shared by four isolates in Guadeloupe.

### Population Structure of *M. tuberculosis* in Martinique

Of 31 isolates studied in Martinique (Figure 5), 19 had an unshared spoligotype, and 12

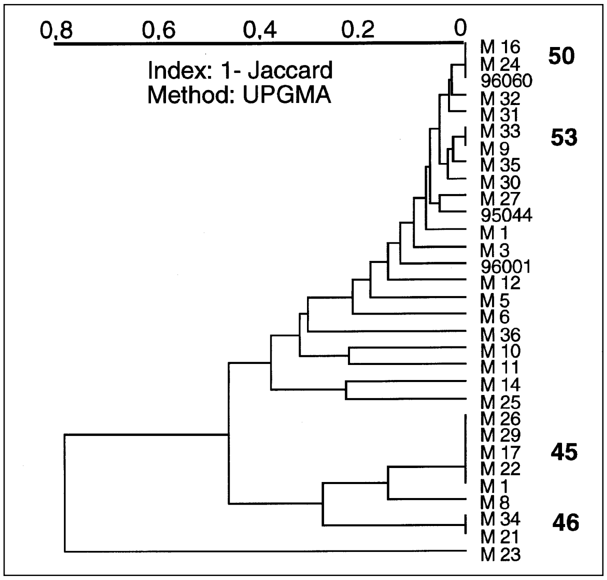

Figure 5. A dendrogram illustrating 31 spoligotyping results of *Mycobacterium tuberculosis* isolates from Martinique. From top to bottom, types 50 and 53, ubiquitous; types 52 and 49 are represented by a single isolate, respectively, M35 and M30, and are ubiquitous; isolates M10 and M7 belong to specific types 17 and 68, respectively found in Guadeloupe and Barbados; M25, belongs to specific type 5 observed in Guadeloupe; type 45, ubiquitous; type 46, ubiquitous; isolate M23 shares type 2, with isolates in Guadeloupe and French Guiana.

shared 4 patterns (pattern 46, 2 isolates; pattern 45, 5 isolates; pattern 53, 2 isolates; pattern 50, 3 isolates). All the clusters in Martinique were also found in Guadeloupe (45, 46, 50, and 53). Pattern 45 is overrepresented in this population, which could suggest active transmission of this clone of tubercle bacilli in Martinique. Other patterns common in Guadeloupe are only poorly represented in Martinique: type 2 (isolate M23), type 5 (isolate M25), and type 17 (isolate M10). Type 5 is specific only to Martinique and Guadeloupe. On the contrary, Martinique shares some patterns with the rest of the world that are not found in Guadeloupe or French Guiana (type 49, isolate M30; type 52, isolate M35). Despite the small sample size of isolates from Martinique, many isolates (clustered or unclustered) share published patterns from the rest of the world (18 of 31 isolates studied).

### Population Structure of *M. tuberculosis* in Other Caribbean Regions

We also investigated 16 additional *M. tuberculosis* isolates (12 from Surinam and 4 from Barbados). Although not representative of the population studied, spoligotyping of these isolates allowed detection of some interesting links (Figure 6). Fourteen unique types and one shared type (pattern 53, two isolates) were found. Among the unclustered isolates, strain 94030 from Surinam matched isolate 94127 from Guadeloupe (type 15), strain 94018 from Surinam matched type 19 from Holland (11), and strain 94034 from Surinam matched isolate 95047 (type 3) from Guadeloupe. Furthermore, two strains from Barbados shared patterns with isolates from other geographic areas: Barb 3 to M7 (type 68) from Martinique and strain Barb 1 to the pattern 61 from France (18).

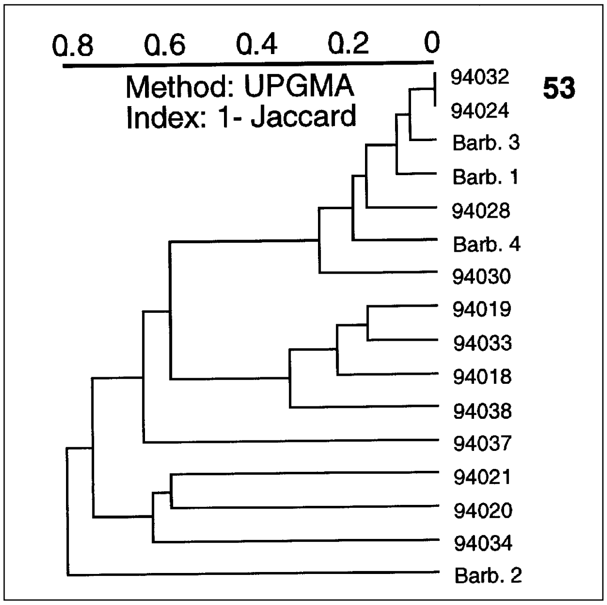

Figure 6. A dendrogram illustrating spoligotyping results of 16 *Mycobacterium tuberculosis* clinical isolates from Barbados and Surinam. From top: type 53, ubiquitous; isolates Barb.3 and 94030 belong to specific types 68 and 15, respectively; isolates 94018, 94020, 94034, and Barb.1 belong to ubiquitous types 19, 1, 3, and 61, respectively.

### Predominant Genotypes and Strain Origin and Transmission

The dendrogram representing the global structure of the population studied (Figure 7) shows the potential historical or epidemiologic interregional flux of *M. tuberculosis* between Caribbean and neighboring Central American regions. Of the 218 isolates, 145 (66.5%) had 29 shared types. Twenty-five isolates were not initially grouped but were clustered only when a dendrogram of all 218 isolates was drawn. These interregional links defined eight new types in the database (patterns 1, 5, 15, 31, 54, 61, 64, and 68) for 15 isolates; the remaining 10 isolates enriched patterns already present (Figure 7). These links, made by finding clonal strains in distant geographic territories, do not necessarily define epidemiologic relationships between these strains (21) but are unlikely to be due to independent genetic evolution. The dendrogram is an indirect picture of the common history of TB spread in this part of the world.

For example, spoligotype 2 was recently proposed to have originated in Latin America (22). Our recent investigations favor this hypothesis as we have traced this pattern in all three territories investigated (Martinique [one isolate]), Guadeloupe [four isolates], and French Guiana [four isolates]). Isolates from all nine patients were further investigated by IS*6110*-RFLP, DRE-PCR, and *Sma*I-PGRS typing (23), and the results confirmed the clonality of this specific cluster. Searching for this genotype in other Latin American countries may help elucidate its origin and distribution.

To define the clonality of the isolates and to be in accordance with the current practice for molecular typing of *M. tuberculosis* using spoligotyping as a first-line method (7, 11, 17, 18), we systematically performed a study of a spoligotyping-defined cluster by a second

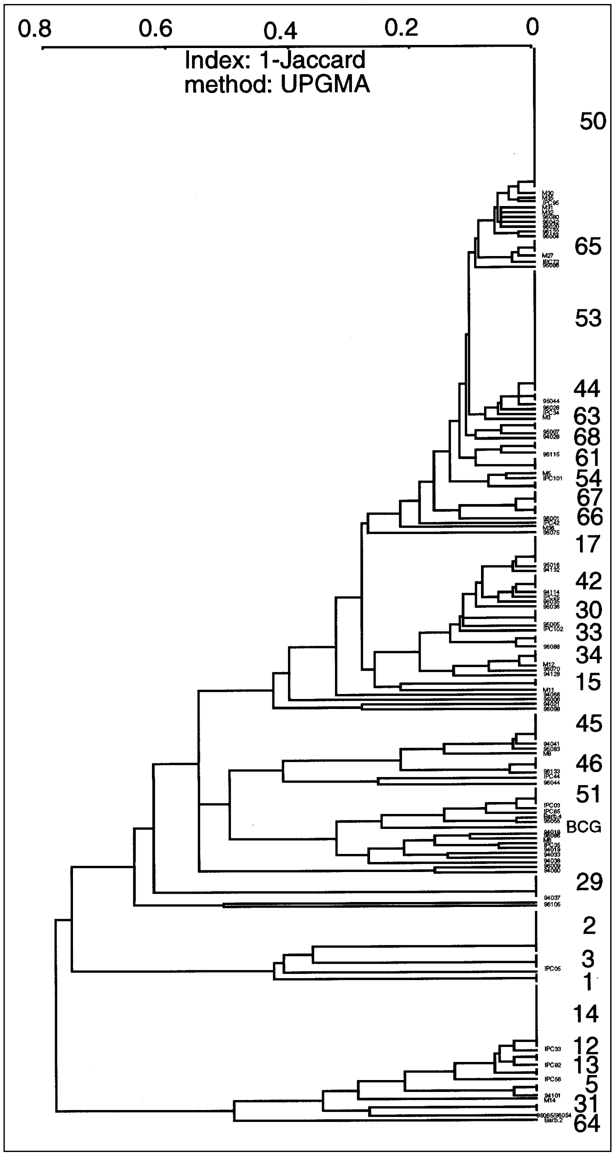

Figure 7. A cumulative dendrogram for the 218 Caribbean isolates of *Mycobacterium tuberculosis*. New types not visible on individual dendrograms can now be observed (types 1, 5, 15, 31, 54, 61, 64, and 68).

method (IS*6110*-RFLP or DRE-PCR). When corroborated by epidemiologic information, the clusters so defined were considered evidence of ongoing active transmission of TB (Table 3). However, in numerous cases, the epidemiologic data were not conclusive, and molecular clonality was not considered direct proof of a link. Thus, despite suspected links in 81 (37%) of 218 of cases on the basis of molecular typing alone, a direct epidemiologic link was demonstrated in 31 (14.2%) of 218 of cases. However, future prospective studies including active case-finding and source tracing may help increase the number of epidemiologically linked cases in our region.

### Distribution of Spoligotypes Bearing Geographic Specificities

To reconstruct the evolutionary tree of *M. tuberculosis* on the basis of 610 different spoligotypes (mostly from the United Kingdom, Holland, France, the Caribbean, and the neighboring Central American region), we performed a similarity search (Figure 2). Although the tree may not reflect the full diversity of spoligotypes, it suggests the existence of distinct families of spoligotypes with geographic specificities (Figure 8). Some strain families may be related to specific populations, geographic regions, and the history of TB spread. For example, the Beijing genotype, which is most divergent in the tree shown in Figure 8 (type 1), would have undergone the most extensive genetic evolution since the origin of *M. tuberculosis*. This information is consistent with the mechanism of evolution of the DR locus, which appears to proceed through losses of direct repeats (24,25).

Although in an unrooted tree, the positions of patterns 50 and 53 correlate well with the isolates most widely represented internationally (116 of 610 isolates studied; 54 of 218 isolates from the Caribbean and neighboring Central American region and 62 of 392 published spoligotypes from different parts of the world) and may constitute a candidate root for the interpretation of distances between isolates.

### Genetic Evolution of Tubercle Bacilli

The genetic evolution of tubercle bacilli is closely associated with the past and present of its host. Consequently, human migration, population mixing, and other sociodemographic factors have long played an important role in the spread and subsequent evolution of the *M. tuberculosis* genome. The insular model of the Caribbean, in which human migration (hence the introduction of the disease) was estimated to occur only approximately 400 years ago, is particularly interesting for discovering conserved strains of tubercle bacilli and for detecting rare *M. tuberculosis* genotypes. In this context, the DR locus is a unique chromosomal

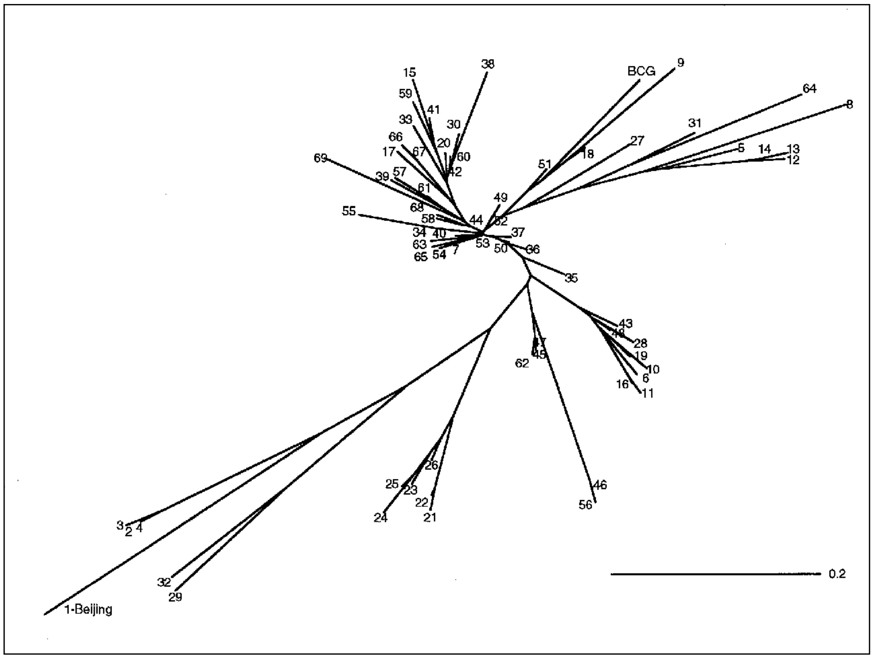

Figure 8. A preliminary phylogenetic tree obtained by the neighbor-joining algorithm on the basis of the 1-Jaccard index (Sj=a/a+c, where a is the number of simultaneously positive characters and c is the number of discrepancies), which is not exhaustive for all existing phylogenetic links. A total of 70 shared spoligotypes were analyzed (69 types shown in Figure 2 and *Mycobacterium bovis* BCG).

region characteristic of the *M. tuberculosis* complex, which shows a high degree of polymorphism that involves homologous recombination and IS-mediated transposition (24). The high degree of internal homology within the DR region of *M. tuberculosis* is likely to favor such genetic rearrangements. Similarly, transposition of IS*6110* is instrumental in generating new subclones of *M. tuberculosis* (26).

Following the principle of Dollo parsimony, which assumes that losses of genes are much more common and likely than independent evolutionary origins (27), the evolutionary process of *M. tuberculosis* can be speculated to have involved the loss of DR repeats. However, the tree in Figure 8 does not perfectly represent all phylogenetic links between isolates as the mechanisms of loss of DR elements (by homologous recombination or replication slippage) could involve simultaneous loss of >1 of the 43 building blocks. The present phylogenetic analysis will be extended to other multiple genetic markers to include variables recently proposed by Sreevatsan et al. (4). However, construction of such trees on the basis of the simultaneous feeding and computer analysis of multiple mycobacterial markers remains cumbersome and constitutes a priority research topic for the studies of *M. tuberculosis* genome evolution.

### Acknowledgments

The authors thank F. Pfaff, L. Schlegel, P. Levett, and P. Prabhakar for sending the cultures for identification to the Institut Pasteur, Guadeloupe, and F. Prudenté, M. Berchel, and K.S. Goh for their expert technical help.

This project was financed by research grants provided by Délégation Générale au Réseau International des Instituts Pasteur et Instituts Associés, Institut Pasteur, Paris, and Fondation Française Raoul Follereau, Paris, France.

Dr. Sola is Chargé de Recherche at the Tuberculosis and Mycobacteria Unit at the Pasteur Institute of Guadeloupe, which is headed by Dr. Rastogi. The authors work closely with public health agencies in Guadeloupe, Martinique, and French Guiana to support the tuberculosis control program. They provide expertise and training on the genetic characterization and laboratory diagnosis of mycobacteria. Their current research interests include molecular diagnostics and epidemiology, drug resistance, and mechanisms of mycobacterial pathogenicity.

### References

1. Cole ST, Brosch R, Parkhill J, Garnier T, Churcher C, Harris D, et al. Deciphering the biology of *Mycobacterium tuberculosis* from the complete genome sequence. Nature 1998;393;537-44.
2. van Helden PD. Bacterial genetics and strain variation. Novartis Foundation Symposium 1998;217:178-94.
3. Small PM, van Embden JDA. Molecular epidemiology of tuberculosis. In: Bloom BR, editor. Tuberculosis: pathogenesis, protection and control. Washington: American Society for Microbiology; 1994, p. 569-82.

4. Sreevatsan S, Pan X, Stockbauer KE, Connell ND, Kreiswirth BN, Whittam TS, et al. Restricted structural gene polymorphism in the *Mycobacterium tuberculosis* complex indicates evolutionarily recent global dissemination. Proc Natl Acad Sci U S A 1997;97:9869-74.
5. van Embden JDA, Cave MD, Crawford JT, Dale JW, Eisenach KD, Gicquel B, et al. Strain identification of *Mycobacterium tuberculosis* by DNA fingerprinting: recommendations for a standardized methodology. J Clin Microbiol 1993;31:406-9.
6. Sola C, Horgen L, Goh KS, Rastogi N. Molecular fingerprinting of *Mycobacterium tuberculosis* on a Caribbean island with IS*6110* and DRr probes. J Clin Microbiol 1997;35:843-6.
7. Sola C, Horgen L, Maïsetti J, Devallois A, Goh KS, Rastogi N. Spoligotyping followed by double-repetitive element PCR as rapid alternative to IS*6110*-fingerprinting for epidemiological studies of tuberculosis. J Clin Microbiol 1998;36:1122-4.
8. Stead WM, Eisenach KD, Cave MD, Beggs ML, Templeton GL, Thoen CO, et al. When did *Mycobacterium tuberculosis* infection first occur in the New World? Am J Respir Crit Care Med 1995;151:1267-8.
9. Sola C, Horgen L, Devallois A, Rastogi N. Combined numerical analysis based on the molecular description of *Mycobacterium tuberculosis* by four-repetitive sequence-based DNA typing sequence. Res Microbiol 1998;149:349-60.
10. Rastogi N, Schlegel L, Pfaff F, Jeanne I, Magnien C, Lajoinie G, et al. La tuberculose dans la Région Antilles-Guyane: situation epidemiologique de 1994 à 1996. Bulletin Epidémiologique Hebdomadaire 1998;11/98:45-7.
11. Kamerbeek J, Schouls L, van Agterveld M, van Soolingen D, Kolk A, Kuijper S, et al. Simultaneous detection and strain differentiation of *Mycobacterium tuberculosis* for diagnosis and epidemiology. J Clin Microbiol 1997;35:907-14.
12. Hermans PWM, van Soolingen D, Bik EM, de Haas PEW, Dale JW, van Embden JDA. Insertion element IS*987* from *Mycobacterium bovis* BCG is located in a hot-spot integration region for insertion elements in *Mycobacterium tuberculosis* complex strains. Infect Immun 1991;59:2695-705.
13. Sneath PHA, Sokal RR. Numerical taxonomy: the principles and practices of classification. San Francisco (CA): W.H. Freeman & Co.; 1973.
14. Saitou N, Nei M. The neighbor-joining method: a new method for reconstructing phylogenetic trees. Mol Biol Evol 1987;4:406-25.
15. Friedman CR, Stoeckle MY, Johnson WD, Riley LW. Double-repetitive-element PCR method for subtyping *Mycobacterium tuberculosis* clinical isolates. J Clin Microbiol 1995;33:1383-4.
16. Poulet S, Cole ST. Characterization of the highly abundant polymorphic GC-rich repetitive sequence (PGRS) present *in Mycobacterium tuberculosis*. Arch Microbiol 1995;163:87-95.
17. Goyal M, Saunders NA, van Embden JDA, Young DB, Shaw RJ. Differentiation of *Mycobacterium tuberculosis* isolates by spoligotyping and IS*6110* restriction fragment length polymorphism. J Clin Microbiol 1997;35:647-51.
18. Goguet de la Salmonière Y, Li HM, Torrea G, Bunschoten A, van Embden JDA, Gicquel B. Evaluation of spoligotyping in a study of the transmission of *Mycobacterium tuberculosis*. J Clin Microbiol 1997;35:2210-4.
19. Douglas JT, Qian L, Montoya JC, Sreevatsan S, Musser JT, van Soolingen D, et al. Detection of a novel family of tuberculosis isolates in the Philippines. 97th general meeting of the American Society for Microbiology, Washington: American Society for Microbiology Press; 1997. p. 572.
20. van Soolingen D, Qian L, de Haas PEW, Douglas JT, Traore H, Portaels F, et al. Predominance of a single genotype of *Mycobacterium tuberculosis* in countries of East Asia. J Clin Microbiol 1995;33:3234-8.
21. Yang Z, Barnes PF, Chaves F, Eidenach KD, Weis SE, Bates JH, et al. Diversity of DNA fingerprints of *Mycobacterium tuberculosis* in the United States. J Clin Microbiol 1998;36:1003-7.
22. Prodinger WM, Pavlic M, Pedroza JC, Allenberger FJ. Tracing of a cluster of tuberculosis infections [abstract U-56]. 98th General Meeting of the American Society for Microbiology, Washington: American Society for Microbiology Press; 1998. p. 504.
23. Jasmer RM, Ponce de Leon A, Hopewell PC, Alarcon RG, Moss AR, Paz A, et al. Tuberculosis in Mexican-born persons in San Francisco: reactivation, acquired infection and transmission. Int J Tuberc Lung Dis 1997;1:536-41.
24. Groenen PMA, Bunschoten AE, van Soolingen D, van Embden JDA. Nature of DNA polymorphism in the direct repeat cluster of *Mycobacterium tuberculosis*; application for strain differentiation by a novel typing method. Mol Microbiol 1993;10:1057-65.
25. van Soolingen D, de Haas PEW, Hermans PWM, Groenen PMA, van Embden JDA. Comparison of various repetitive DNA elements as genetic markers for strain differentiation and epidemiology of *Mycobacterium tuberculosis*. J Clin Microbiol 1993;31:1987-95.
26. Bifani PJ, Plykaitis BB, Kapur V, Stockbauer K, Pan X, Lutfey ML, et al. Origin and interstate spread of a New York City multidrug-resistant *Mycobacterium* clone family. JAMA 1996;275:452-7.
27. Meyer A. In: Harvey PH, Leigh Brown AJ, Maynard Smith J, editors. New uses for new phylogenies. London: Oxford University Press; 1996. p. 322-40.
